# Pennsylvania State Dental Society

**Published:** 1889-10

**Authors:** F. S. Bassett

**Affiliations:** Philadelphia


### Reports of Society Meetings.

---

### PENNSYLVANIA STATE DENTAL SOCIETY.—(*Continued.*)

### THE USE OF THE MATRIX WITH PROXIMAL AMALGAM FILLINGS.

BY F. S. BASSETT, D.D.S., PHILADELPHIA.

For many years various forms of matrix have been used to facilitate the introduction of gold into proximate cavities, and recently its use has become somewhat more extended in connection with amalgam in similar places. With the hope of inducing still more of the profession to adopt this plan, I bring this subject before you, feeling assured that, as with myself, its use will be attended with great satisfaction. Some may question the advisability of consuming any time for the purpose of fixing a matrix for so short an operation as filling a cavity with amalgam, but with me the advantages have been so great that I almost always use the matrix for proximate fillings. I question, however, whether any time is really lost by fixing a matrix, even for fillings of ordinary difficulty; and in very extensive cavities, reaching to or beyond the gum, much time is certainly gained by this method.

An examination of the proximate amalgam fillings which come under our notice is apt to reveal two general defects,—an insufficiency of contour, and an excess of filling at the cervical border. I am an earnest advocate of restoring normal contour to all teeth which require filling upon their proximate surfaces, for my observations have proven to me most conclusively that such teeth are far less liable to recurring decay than if left with flat surfaces. In addition, there is the greater stability to the whole arch, to say nothing of the greater comfort to the patient. Of all uncomfortable conditions to which the teeth are at times subjected, I know of none greater than when amalgam fillings project beyond the cervical border of a proximate cavity. Such places afford a convenient pocket for the lodgement of particles of food, which, if not soon removed, cause further decay at or below the border of the gum; and this is frequently not discovered until the pulp is nearly or quite exposed.

If attempts are made by the patient to remove the *débris* from such places, continued irritation to the gum is produced, and a great amount of discomfort occasioned. It was for the correction of this defect that I first began using the matrix, but the results obtained at other points of the filling have been so satisfactory that I now look upon it as a necessary aid to success in very difficult and extensive cavities.

Many forms of matrix have been devised, each having some particular claim to merit; but the form which I use is exceedingly simple, and answers the purpose perfectly. It consists of a piece of German-silver plate about one-thirtieth of an inch in thickness, cut and bent in the proper form for the case in hand. In an examination of the proximate surfaces of molars and bicuspids we usually find that the form of surface changes from convex upon the largest part of the crown to concave at or below the gum-line. Here arises the necessity for a matrix which can be readily adapted to the borders of the cavity, and also give the requisite contour to the filling. The German-silver matrix can be easily bent to fit any form of surface and yet is sufficiently rigid when wedged in place to bear all the pressure necessary for the introduction of the filling.

I will not dwell at any length in this paper on the necessity for the thorough preparation of the cavity, as it is well understood that to attain success thoroughness is as indispensable with amalgam as with gold. Particular attention must be given to bevelling the borders, since amalgam cannot be brought to a knife-edge as is often done with gold, for it will soon crack away and leave the filling imperfect. I usually try to shape the cavity so that the line of junction of tooth and filling will be at right-angles to the surface. Then there is the least danger of enamel and amalgam chipping away.

Before wedging the matrix in place the inner surface should be polished, which can be quickly done with pumice-powder upon a few fibres of cotton wound about a wooden mandrel in the engine hand-piece. The cases which are ordinarily the most difficult are by the use of the matrix reduced to simple cavities, which can be rapidly and accurately filled. With the amalgam mixed to a medium plastic condition, it can be pushed into all parts of the cavity with a firm pressure; and by making use of pellets of Japanese bibulous paper, as suggested by Dr. Bonwill, pressed firmly upon the surface of the amalgam it is packed into a homogeneous mass, and the surplus mercury brought to the surface, whence it can be readily removed. After the cavity is filled and the matrix removed, the filling will be of the proper form without any concern as to the troublesome cervical edge, usually so difficult to shape accurately.

I invariably "finish" the filling at a subsequent sitting, after it has thoroughly hardened, much in the same manner as I finish a gold filling, with polishing tapes, powders, etc. The surface then remains in an agreeably smooth condition and the edges are brought out clearly and well-defined. The filling, then, besides serving in the highest degree the conservation of the tooth, has all the artistic excellence possible to attain. I fully believe that many extensive and difficult cavities in molars and even in bicuspids can be filled with more credit to the operator and satisfaction to the patient with amalgam than with gold. The great fatigue and possible prostration of long operations are thus avoided.

Another point in the consideration of the use of the matrix with proximate fillings of amalgam is that the rubber dam is seldom required. Even in mouths where the saliva is unusually abundant the cavity can be kept dry long enough to cover the whole surface with a layer of amalgam, after which the moisture will not seriously interfere with the successful completion of the operation.

### DISCUSSION ON DR. F. S. BASSETT'S PAPER ON "THE USE OF THE MATRIX WITH PROXIMAL AMALGAM FILLINGS."

*Dr. Jos. Head*, Philadelphia.—Does Dr. Bassett ever put gold on top of the amalgam?

*Dr. Frank S. Bassett.*—I do not, but it is entirely feasible to do so. I do not combine the two metals, because I think the amalgam answers all necessary purposes, except in some places where the exposed part shows too much. Then a gold face would be good.

*Dr. L. A. Faught*, Philadelphia.—I am very much interested in the practice of using matrices in making proximal fillings of amalgam. I have lately begun their use in this relationship. I cannot say that I am successful in all instances, for I find that there are some difficulties at times. I have frequently found the matrix difficult to remove without detaching portions of the filling. It may be due to my faulty adjustment. I would like to ask the members regarding their experiences, for when I get my filling sufficiently hard, in removing the matrix I not unfrequently get into trouble. If I leave it soft I know I am in trouble. I also have a suspicion as to whether I do not drive the mercury into the edges of the filling. These are points of practical significance to us, and they are at present unsettled in my experience. I mention them so as to start the ball of discussion going and to get practical experience.

*Dr. F. S. Bassett*, Philadelphia.—In reply to one objection of Dr. Faught's, that of displacing the filling in removing the matrix,—the thin German-silver matrix described can be bent into a straight piece, and pulled out sideways with pliers, without any injury to the filling. I have very little difficulty on that point, and if the matrix is wedged into position firmly, after removing your wedge, you have a little additional space for removing the matrix.

Regarding the fear of having a surplus accumulation of mercury along the edges and making the filling soft, I do not think there is any trouble, for it always seems to work to the surface for me. If it works out it is in globules, which leaves the edges hard.

*Dr. F. H. Abel*, Erie.—I have not had any trouble with the mercury in that way yet.

*Dr. C. V. Kratzer*, Reading.—Will Dr. Bassett mention whether he uses any particular matrix.

*Dr. F. S. Bassett.*—I make it for each particular case by using a piece of German-silver cut into the shape I want it.

*Dr. C. V. Kratzer.*—Is it retained without any appliances?

*Dr. F. S. Bassett.*—No; there must be something to wedge it in place. It is preferable to have enough space to contour readily.

*Dr. G. L. Robb*, Huntingdon.—I never think of filling a proximal cavity without a matrix. There is no danger in putting it on, and no danger in removing it. You cannot destroy the filling at the cervical portion of the tooth, because it will work up itself. I always polish an amalgam filling afterwards, if possible, using a disk or polishing-strip on it. I do not know about the German-silver strip spoken of; I use the Miller matrix. All I have to do is to loosen the screw-press a little in the space between it and the next tooth, and it is taken off. I pass up silk in order to be sure that there may be no projections, and that it may be perfectly smooth.

*Dr. W. B. Miller*, Altoona.—I have been styled a "matrix crank." As all know, I have devised several forms of matrices, and not once in six months do I fill a tooth—not even with cement—without a matrix, and I think that every man who does not use the appliance is throwing away half his life. I approve of what Dr. Bassett says, of course, and give a man credit for using the matrix. There are forms of teeth that will require a special matrix made for that purpose.

In reference to breaking off a portion of the contour of fillings,

I have had very little difficulty in that respect. The matrices I use are made of very thin steel, and I always manage to have room enough to place back of them an additional thickness of steel; and after the filling is in position and thoroughly condensed, I remove that little addition of thin steel, and it gives me space to take out the band. The advantage of having a matrix with an adjustable screw is that you can use it over and over again.

*Dr. C. V. Kratzer.*—In my experience it does not matter very much what you use for proximal fillings, but it is essential to have some kind of matrix. It is a great saving of labor, especially where two amalgam fillings approximate, and in large separations it seems to me it would be almost impossible to put in two amalgam fillings perfectly without a matrix of some kind. I use matrices made of silver strips, having acquired the habit of passing the strip clear around the tooth and holding the end with the left hand, and also holding the amalgam director with the same hand. I also use thin steel strips and thin pieces of file—separating file. Then, of course, the filling requires some trimming afterwards, especially at the cervical edge.

*Dr. W. B. Miller.*—I find a little objection to the plan of Dr. Bassett,—that of wedging the matrix into position. In case decay has gone all the way down, I think we ought to have a surplus of material and cut it down afterwards. If you fill to a flush you have nothing to work on afterwards.

*Dr. H. E. Roberts*, Philadelphia.—The continued pressure in inserting the filling will cause the matrix to bulge a trifle, and thus secure the desired end.

*Dr. F. S. Bassett.*—Regarding the use of a piece of file, and then having to trim the cervical edge, I think there is a great advantage in getting your matrix under the cervical edge in the first place, because it is hard to trim an amalgam filling afterwards. If it is soft you are apt to take too much, and if it is hard it is pretty difficult to remove at the cervical edge, therefore I want to accomplish this in inserting the filling, if possible.

*Dr. L. A. Faught.*—That is just where the difficulty comes in,—that of the mercury going to the side of the cavity. If we allow the matrix to bulge, the filling projects and leaves the amalgam at the side of the cavity in a more or less softened condition. If you make your matrix tight, the mercury will come to the surface.

*Dr. Jos. R. C. Ward.*—I suppose I stand alone in that I do not use matrices, at least not to any great extent. I think we are apt to depend too much on the matrix to hold the filling in position while inserting it, and the result is the filling will not last sufficiently. With the matrix we do not take the same care to shape the cavity and hold the filling as we do without it, and I have had very little trouble in using amalgam except in distal surfaces of second and third molars, when on account of the want of room in removing the rubber dam, I remove the filling. That, perhaps, might be avoided by using a matrix. I use a filling material that sets hard at the time,—Flagg's Standard Alloy: it becomes hard in a few moments. I keep the patient in the chair until hardness is effected. For that reason I have not used the matrix very much. The only case where I could use it effectively would be in the large cavities on the distal surfaces of back teeth. I have had very little trouble in finishing the cervical edges of fillings without the matrix, simply because while the amalgam is getting hard I can do the work.

*Dr. W. B. Miller.*—Why use a matrix in these particular cases?

*Dr. Jos. R. C. Ward.*—It is impossible sometimes to get the rubber dam up sufficiently far to clear the tooth where it is decayed. We can press up a matrix to a degree we could not the rubber dam.

*Dr. W. B. Miller.*—Will you please state how you fill two proximate cavities,—say a molar and bicuspid,—how you would condense and finish?

*Dr. Jos. R. C. Ward.*—First prepare the cavities; fill one, let that get perfectly hard, and then fill the other.

*Dr. Jas. S. King.*—Do you not find the clamp remove the filling more than the rubber dam?

*Dr. Jos. R. C. Ward.*—I think, perhaps, it does.

*Dr. Alonzo Boice*, Philadelphia.—I was unfortunately away when the paper was read, and hence did not get the benefit of its contents. Merely because you have failures,—perhaps due to faulty manipulation, or not using the proper kind of amalgam,—you say it is the fault of the filling. It is the operator. We fill with amalgam and gold, and why? They will tell you it saves the teeth. Some say the best method of inserting an amalgam filling is to place it in the cavity and finish the next day. It can be done as well ten minutes after the insertion as ten days afterwards.

Regarding the method of getting amalgam away from the surface, if you make an opening between the walls of the matrix you must force the free mercury through. The filling with amalgam should be attended with as much care as filling with gold, and be finished so hard that you can pound on it. What kind of amalgam?

Some are better than others, but I care not what you use. I use probably more Standard than any other.

*Dr. Jas. S. King.*—The doctor made an assertion that some amalgams were better than others. Why are they better?

*Dr. Alonzo Boice.*—I meant that I could work them better than others. I use Standard, but I do not say it has virtues over all others.

*Dr. Jos. R. C. Ward.*—One reason why I like the Standard alloy is, it is a fine grade, and in working it up it becomes smooth in the fingers. It sets quickly, and the reason I like it best is I can work it better than other makes.

*Dr. G. L. Robb.*—Dr. Boice says he can pound on an amalgam filling in ten minutes.

*Dr. Boice.*—No, I did not mean that.

*Dr. Robb.*—I would say do not bite on it.

*Dr. Boice.*—I tell the patient to go out and eat his dinner, and pay no attention to the filling.

*Dr. Jas. S. King.*—My friend Ward speaks of smoothness in amalgam. There is a principle which produces that. Dr. Boice speaks of Standard alloy. This is richer in silver than any other make I know of, and the percentage of silver makes the condition he speaks of. Take the experiments of Hitchcock and Bogue, some time ago published by the Odontological Society, of New York. Many of the amalgams they tested shrank, and the highest percentage of silver they found an amalgam to contain was fifty-six. Most of them ran from thirty-one to fifty-six. In an article in a Western journal it is stated that an amalgam composed of eighty per cent. of silver is free from shrinkage. It is fine in texture and contains about eighty per cent. of silver, fifteen of tin, three of platinum, and two of zinc. What does the zinc and the other metals do? In the experiments of Hitchcock and Bogue the tin accounted for the shrinkage, because in their report Dr. Hitchcock stated that an amalgam composed of silver expanded twenty degrees. It showed the contraction was not in the silver, but in the tin. Then the question was, Why did they not experiment on amalgam containing a greater amount of silver? From experiments like these I am very clearly convinced that an amalgam should contain seventy-five to eighty per cent. of silver to avoid contraction, and if there is a small amount of tin, as well as platinum, it adds strength and toughness. The object of the zinc is to prevent discoloration. It acts on silver like zinc on iron. Two per cent. will not produce in the mouth a galvanic cement that will be perceptible to any extent.

I never use matrices. Believe I have only used one once, and yet I think I have done as fair work with amalgam as some of my friends. We must get the point between contraction and expansion. When you get that balance you want something that will prevent discoloration, and something that will give edge and strength, and these are the reasons why I use an amalgam composed of eighty parts of silver, fifteen parts of tin, three parts of platinum, and two parts of zinc.

*Dr. J. R. C. Ward.*—Don't you have greater discoloration for the greater amount of silver?

*Dr. J. S. King.*—The zinc overcomes the tendency towards discoloration. If we could use forty per cent. of zinc without a galvanic current being produced it would be better. That amount might produce conditions that would result in a degeneration of the filling, which a less percentage of zinc might not produce.

*Dr. C. S. Beck.*—I would like to know what gives the amalgam this soft velvety feeling.

*Dr. Ward.*—Dr. King says it is due to silver.

*Dr. Beck.*—I doubt that. I think it is due to the tin. The best amalgam I use is Welch's alloy, and I use to some extent copper amalgam. I think in children's teeth it is just the thing. I do not see why Dr. King objects to the matrix.

*Dr. J. S. King.*—I do not object to the matrix. I have used it only in one case. The velvety condition I cannot account for precisely. In mixing up an amalgam, if I wash it with alcohol, I get the desired quality.

*Dr. Beck.*—I never wash an alloy; but the velvety feeling of the amalgam is due to tin and not to silver.

*Dr. King.*—If due to tin, why not use an amalgam that has a larger per cent. of tin in it?

*Dr. Faught.*—My personal belief is that where you increase the percentage of silver you have to increase the proportion of mercury, and you thus get the velvety feeling. I do not think it is reliable when as high as eighty per cent. of silver is used.

*Dr. Boyce.*—Are we not drifting in our discussion? The subject is not amalgam filling. I suppose Dr. Basset merely advocated the use of a matrix to show how we could do better work. I think many amalgam fillings are inserted with the idea that they are cheap, and less care is taken in its making than with gold. I am convinced that if amalgam is worked as carefully as gold it is a first-class article.

Subject passed.

DISCUSSION ON DR. GEO. F. ROOT'S PAPER ON THE PREPARATION AND FILLING OF ROOT CANALS.[1]

*Dr. Jos. Head.*—Dr. Root said he hoped some dentist would conceive of some filling material that would combine all the advantages of oxychloride of zinc, gutta-percha, and cotton, and yet have none of their disadvantages. The perfect root-canal filling I hold is that which thoroughly protects the canal from the entrance of septic matter, and prevents the dentine around the canal from containing germs that may in time work their way into the canal, and that filling material I think has been found. It is "carbolized cosmoline upon cotton." After having read the paper I did to-day, I should be very reticent about coming forward without any facts. Gentlemen, I think it is only fair that I show you these specimens I have at hand.

1. A right central; canal was enlarged and carefully filled with gutta-percha.

2. Another central; canal drilled out, dried, and filled with carbolized cosmoline and cotton.

3. A central; canal enlarged, filled with oxychloride of zinc.

I then protected the cavities in the crowns of the teeth, so that no leakage would be possible from that source. After this I dropped the teeth in analine ink, and left them to remain for twenty-five days. At the end of that time I took the teeth out, wiped them dry, and exposed the canals. I thought that after this experiment the canal which would be free from the stain of the red analine ink would contain the perfect filling material. I found that the gutta-percha, as you can see, leaked badly, as much leakage occurring through the dental tubuli as through the apical foramen. The oxychloride filling is also badly stained. In the tooth filled with cosmoline, the dentine, as you can see, has a semi-translucent greasy appearance, but not the slightest trace of ink. The ink has not only been excluded from the canal, but kept absolutely out of the dentine. It may be said I used dry teeth here, and the teeth in the mouth are wet. That is true. What we wish is a filling material that will keep the canal dry, and so I have experimented on moist teeth, and I found that the carbolized cosmoline, when the canal was partially dried, did not permeate the entire structure of the dentine, but only in a circular ring round the canal; but it prevented leakage in the same way. The canal was as strongly protected in this instance as if the entire mass had been filled with

---

[1] Paper printed on page 584 of present issue.

cosmoline. I was at first surprised in experimenting with moist teeth that I filled with gutta-percha and oxychloride of zinc, because I found that the red ink had not penetrated the dentine, when soaked for twenty-five days, nearly so thoroughly as when dry teeth were used, and that the filling material was only stained at the top; but that was easily explained, because when a moist tooth is used the dental tubuli, through which the leakage in part takes place, are already filled with a colorless fluid, and before the ink, in which the tooth is placed, can reach the canal it must completely drive that colorlesss fluid out or work through by means of osmosis. That must take a very long time, and so, although in the first tooth the same amount of leakage occurs, it does not appear, because the filling is colorless; while, when I use a dry tooth, the same amount of moisture enters the tooth, and is in the same relative position to the filling materials, but its presence is at once made known by means of the red ink. Some of my friends have claimed that oxychloride of zinc is just as good, because, when moist chloride of zinc is introduced into a root canal, the dental tubules become filled with zinc chloride, and therefore are in an antiseptic state; but chloride of zinc does give rise to offensive vapors, which cause alveolar abscess.

*Dr. Kirk.*—How do you know?

*Dr. Head.*—That is my opinion. I am now speaking before this assembly for the purpose of laying all the facts that I possess before them, not for the purpose of giving my opinion, or forcing any method upon them, but in order that they may philosophically judge for themselves.

*Dr. W. B. Miller.*—The essayist forgot to mention one good filling,—sandarach, varnish, and cotton. I think if he places one in and undertakes to take it out some time afterwards he will find he has a perfect root-filling.

*Dr. Jos. R. C. Ward.*—Regarding the discoloration, could not the density of the teeth have an influence on this? Would not the size of the foramen also have an influence? In taking several teeth it is impossible to get them all of the same density; analine would act more easily upon one than the other, also, you may use more care in filling with one material than with another. This, of course, will detract very much from the cases presented, simply because one tooth may be denser than the other.

*Dr. Head.*—This brings out the point in question,—the liability of error in experiments. The best thing to do, then, is to verify the matter.

*Dr. W. B. Miller.*—Dr. Head, I want to call your attention to a case where the permeation of cosmoline through the entire tooth-structure caused me considerable annoyance and inconvenience. Several years ago I had a patient, a young girl about fifteen years of age, who came to me with a left superior central incisor,—anterior and posterior proximal surfaces badly decayed. It was a large and expensive operation. I filled the canal with cotton. Less than three months ago the patient returned with the tooth terribly discolored, but the gold work I had put in was as nice as I ever performed. I could not account for it, and had to cut the crown off and put on a Logan tooth crown. I was ashamed of it. Any gentleman who fills a tooth canal with cosmoline, I want him to be a little bit careful that he does not have a discolored tooth.

*Dr. Head.*—I am much obliged for your warning. I wish you would also carry on the experiment, not for me, but for the profession at large; that you take a tooth, say a central that can be easily filled; put the root of the tooth in plaster; moisten the plaster so that the tooth will be always moist; bur out the canal to the best of your ability, and put in cosmoline, and see if you can get the cosmoline to permeate the entire tooth-structure. It will be only the dentine that is thus affected.

*Dr. G. W. Klump.*—What do you use on the cosmoline?

*Dr. Head.*—I place cotton and cosmoline up three-quarters the length of the canal. On top of this I place a cement filling, having wiped out the excess of cosmoline. Over the cement I put gold.

*Dr. G. W. Klump.*—I should think there would be danger of getting your cosmoline on the gold.

*Dr. Head.*—I line the cavity because I think if the dentine could be made water-tight it would be less liable to decay. I dry out thoroughly and have the cosmoline soak into the tubules a short distance around the cavity. then wipe out thoroughly the excess. In packing the gold I put one piece over the dentine, and then lay piece on piece. The first piece only comes in contact with tooth-substance, and forms a covering to the dentine.

Subject passed.

(To be continued.)

